# Leveraging population-scale proteomic data with deep learning for head and neck cancer detection in saliva

**DOI:** 10.1038/s41746-026-02658-7

**Published:** 2026-05-02

**Authors:** Anza Shakeel, Samuel W. D. Merriel, Joel Smith, A. Stephen McGough, Matthew Suderman, Zahraa S. Abdallah, Paul Yousefi

**Affiliations:** 1 MRC Integrative Epidemiology Unit, University of Bristol, Bristol, UK; 2 Centre for Primary Care & Health Services Research, University of Manchester, Manchester, UK; 3 College of Medicine and Health, University of Exeter, Exeter, UK; 4 Royal Devon University Healthcare NHS Foundation Trust, Exeter, UK; 5 School of Computing, Newcastle University, Newcastle, UK; 6 NIHR Bristol Biomedical Research Center, University Hospitals Bristol and Weston NHS Foundation Trust and University of Bristol, Bristol, UK; 7 School of Engineering Mathematics and Technology, University of Bristol, Bristol, UK

**Keywords:** Biomarkers, Cancer, Computational biology and bioinformatics, Oncology

## Abstract

Identifying robust biomarkers for early cancer detection remains challenging, particularly when working with limited or heterogeneous datasets. Here, we present a proof-of-concept deep learning framework for cancer classification using blood-based proteomic profiles. Our approach leverages sample type transfer and synthetic data augmentation to improve performance and generalization across sample types. Models were trained on plasma proteome data from 13,208 pan-cancer cases and 39,806 controls in the UK Biobank. To address class imbalance and enrich the feature space, a convolutional neural network (CNN-Synth) was trained to detect cancer cases using data augmented with synthetic pan-cancer samples generated via a variational autoencoder. Performance was evaluated in an independent saliva-based dataset from a head and neck cancer case-control study (n = 156). CNN-Synth (AUC = 0.88) surpassed models trained without synthetic data (AUC ≤ 0.77). SHapley Additive explanations identified well-known cancer markers as key features. These results highlight the use of sample type transfer and synthetic data augmentation, with further validation needed.

## Introduction

Proteomic biomarkers offer a promising avenue for non-invasive cancer detection, with potential applications across early diagnosis, risk stratification, and treatment monitoring. Measurable in blood, saliva, or tissue, proteins can reflect dynamic changes in tumor biology, making them valuable candidates for identifying cancers when they are still treatable. However, the discovery of robust proteomic biomarkers is often constrained by small case numbers, high-dimensional feature spaces prone to overfitting, and biological variability across cancer types and tissues^1–4^. For example, study recruitment for rare cancers, like head and neck cancer, is difficult because of their low incidence at the population scale. However, biomarker development for early detection is critical due to their rising incidence and disproportionate contribution to cancer mortality due to late-stage diagnosis^5,6^. Despite these challenges, population-scale proteomic data has demonstrated strong potential for cancer detection^1,2,7^. The UK Biobank’s (UKB) proteomic resource—comprising 2,941 proteins measured in plasma from 53,014 individuals—provides a powerful foundation for biomarker discovery and disease mechanism studies^8^.

Machine learning approaches are well-suited to extract disease-relevant signal from high-dimensional molecular datasets, including those with proteomic measures. Already, a wide range of machine learning techniques have been applied to predict disease risk and patient outcomes^9–11^. Deep learning approaches, particularly convolutional neural networks (CNNs), have expanded predictive capabilities by learning hierarchical feature representations from high-dimensional data^12^. Although CNNs are best known for applications in medical imaging, they are beginning to be applied to omic data for disease prediction^11,13–18^.

One very useful feature of deep learning models is their ability to generalize beyond the specific task for which they were trained. For example, CNNs trained in specific disease contexts have been found to capture variation relevant to multiple diseases, likely due to their capacity to characterize general biological relationships among molecular traits. This property can be leveraged through a transfer learning strategy in which knowledge learned from a source dataset is applied to a related target dataset without additional model adaptation. In this study, we refer to this strategy as sample type transfer, where a model trained on biomarkers derived from one biological sample type is directly evaluated on another, enabling assessment of how well the learned molecular representations generalize across biological sample types. In this context, models trained for one outcome can be used to predict or analyze less common outcomes that lack sufficient training data^11,19,20^. Alternatively, another class of generative deep learning models, called variational autoencoders (VAEs), is specifically developed with the general aim of capturing low-dimensional representations of complex relationships between features. These models have been widely applied not only to generate synthetic data, for example, to address class imbalance in the context of rare cancers^11,21,22^—but also for dimensionality reduction and unsupervised clustering of high-dimensional biological data. In particular, VAEs have been used to uncover biologically meaningful protein modules from large-scale plasma proteomic datasets, as demonstrated by ref. ^15^.

In this study, we apply VAE-based data simulation to address class imbalance and sample type transfer across outcomes and tissue types for HNC detection. We frame this approach as sample type transfer because our method is based on a CNN model pre-trained on blood plasma protein abundance data from a pan-cancer case-control study within the large UK Biobank (UKB) cohort, which includes samples from individuals with HNC. The trained model is then evaluated on saliva samples from a smaller HNC-specific case-control study (SensOrPass). When training, we employ a VAE to generate synthetic cancer protein profiles to augment the number of cases. We show that the resulting model, CNN-Synth, performs better than non-neural-network approaches for distinguishing between HNC cases and controls. SHapley Additive exPlanations (SHAP)^23^ for this model highlight a critical role for a set of well-known cancer biomarkers. In addition to direct sample type transfer, we further investigated whether supervised adaptation to the target tissue (saliva) could improve performance. To this end, the pretrained CNN-Synth was fine-tuned on the SensOrPass dataset using repeated stratified cross-validation. This allowed us to compare direct sample type transfer with transfer learning that incorporates target-tissue adaptation. Finally, SHapley Additive exPlanations (SHAP)^23^ were used to interpret the CNN models, further supporting the importance of these biomarkers..

## Results

**Fig. 1:**
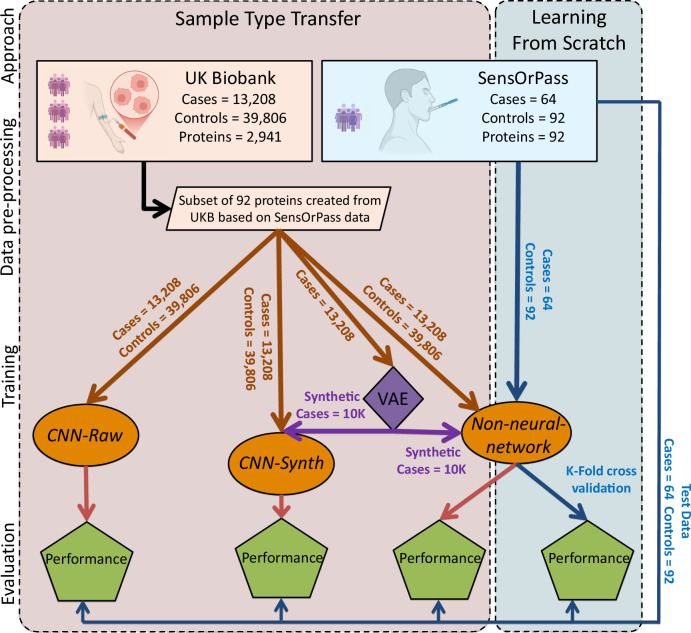
Overview of the applied deep learning pipeline for cancer detection using proteomic data. The left panel illustrates our sample type transfer approach: CNN-Raw and non-neural-network models trained on UKB data using 92 selected protein measurements, as well as CNN-Synth and non-neural-network models trained on a combined dataset of UKB and synthetic cancer cases generated by a variational autoencoder (VAE). These models are evaluated on the SensOrPass dataset (as per the sample type transfer). The right panel represents 'Learning From Scratch' where Non-neural-network models (e.g., LR-elasticnet, LDA, KNN, CART, NB, SVM and XGBoost) were trained and evaluated directly on the SensOrPass cohort using 10-fold cross-validation. ML machine learning, UKB UK Biobank, VAE variational autoencoder, LR-elasticnet logistic regression, LDA linear discriminant analysis, KNN k-nearest neighbors, CART decision trees, NB Gaussian naïve Bayes, SVM support vector machines and XGBoost eXtreme gradient boosting.

With the aim of sample type cancer prediction and proteome biomarker discovery, we trained CNN pan-cancer classification models using the UKB plasma proteome data and evaluated these models on the SensOrPass saliva proteome dataset. An overall summary of our modeling workflow and the data used is provided in Fig. 1 and Table 1, respectively.

**Table 1:** Details of the SensOrPass study (saliva samples collected for this study) and UKB populations with proteome data

Study populations	Sample size *n*	Age mean (SD)	Disease Status	Case *n* (%)	Control *n* (%)	Sample type	Ethnicity
UK Biobank (Training data)	53,014	56.8 (8.2)	Pan-cancer	13,208 (24.9%)	39,806 (75.1%)	Plasma	≈90% White British
SensOrPass (Test data)	156	63.8 (9.6)	Head and Neck Cancer	64 (41.02%)	92 (58.9%)	Saliva	98.9% White British

### Training a VAE of cancer-specific protein abundance

To potentially mitigate the negative impact of class imbalance on model performance, we explored the utility of generative deep learning to synthesize case data. For this, we trained a VAE on data from 13,208 UKB cancer cases and used it to generate 10,000 synthetic pan-cancer samples (see Methods Section “Variational autoencoder (VAE) of cancer-specific protein abundance”). These simulated cases were added to the original training set, increasing it to 23, 208 to improve class balance (Fig. 1).

The VAE effectively captured key marginal biological patterns between the real and synthetic data (Supplementary Fig. 1a–c). PCA and t-SNE visualizations show that the simulated pan-cancer samples broadly overlap with the distributions of both the UKB and SensOrPass cancer cases. Quantitatively, Kolmogorov-Smirnov (KS) statistics for all 92 biomarkers are all below 0.34 (Supplementary Fig. 1d), and per-feature discrimination AUCs obtained from univariate logistic regression models are tightly centered around 0.5 (mean = 0.496, SD = 0.016), indicating close alignment of marginal biomarker distributions. Although the random forest classifier could distinguish real from synthetic samples (AUC ≈ 0.79), feature-level importance values remained uniformly low, suggesting that differences arise from higher-order correlations rather than individual markers (Supplementary Fig. 1e–g). The synthetic samples were therefore used to augment the minority (cancer) class during CNN training, providing biologically coherent examples for class-imbalance correction.

Although it would have been possible to generate a larger number of synthetic samples to further mitigate the impact of class imbalance, we deliberately limited the number to 10,000 synthetic cancer cases. This quantity represents approximately 43% of the cancer-positive class in the training data and was selected to achieve a balance between improving class representation and minimizing the risk of overfitting.

### Classifying cancer with protein-based CNN models

**Fig. 2:**
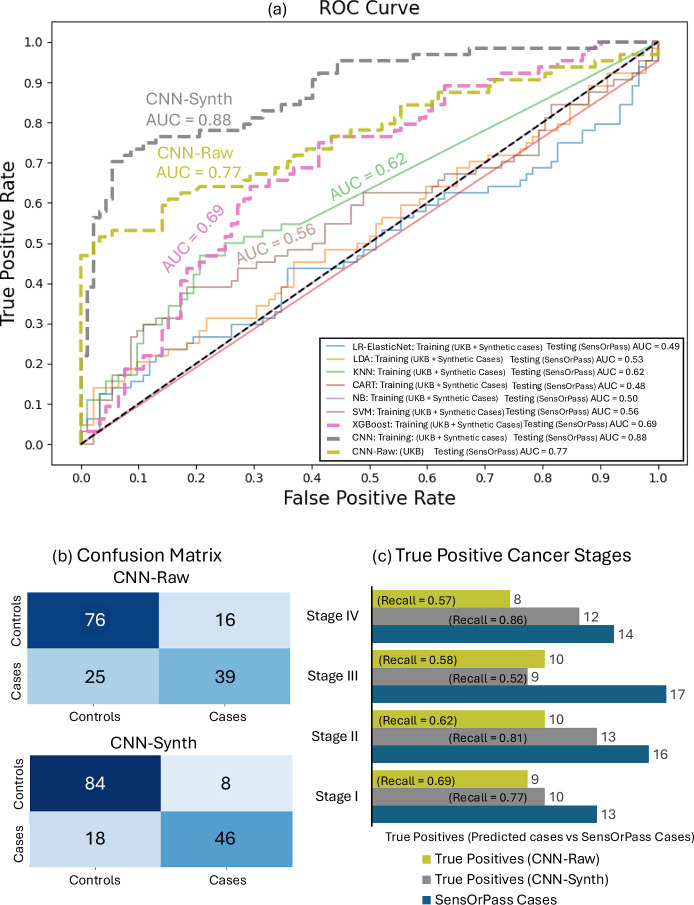
Performance evaluation of sample type transfer approach. **a** ROC plots of all models evaluated in SensOrPass. **b** Confusion matrices of both CNN-Raw and CNN-Synth models, showing improved classification accuracy across cases and controls when synthetic data is included in training. **c** Bar plot of true positives by TNM staging for CNN-Raw (green) and CNN-Synth (gray). Recall (sensitivity), calculated as the proportion of true positives relative to the total number of cases per stage, is shown in brackets on each bar. No information of staging was available for 4 out of 64 HNC cases in the SensOrPass study. ROC receiver operating characteristic, AUC area under the curve.

We trained two CNN pan-cancer classification models in UKB (Figs. 1 and 4b). The first model, called CNN-Synth, was trained on data from UKB cases and controls along with 10,000 synthesized cases. The second, CNN-Raw, was trained only on UKB cases and controls to evaluate the impact of the synthesized cases on CNN-Synth. Due to the small size of the SensOrPass dataset (*n* = 156, 64 cases), which was used exclusively for testing, training a deep learning model solely on this cohort would result in severe overfitting. Therefore, both CNN models were trained on UKB data, with CNN-Synth additionally augmented by synthetic samples, and subsequently evaluated on the independent SensOrPass cohort. Evaluation of both models in SensOrPass revealed that the addition of synthetic samples in the CNN-Synth training dataset improved most performance metrics by a large margin (Table 2 and Fig. 2a). Compared to CNN-Raw, there is a decrease in miss-classification both for controls and cases, false positives decreased from 17 to 11 and false negatives from 30 to 21 (Fig. 2b).

**Table 2:** The table reports the Area Under the Curve (AUC), F1-score, Precision, and Recall for all models across three evaluation scenarios: (i) training and testing on the SensOrPass dataset, (ii) direct sample type transfer (training on UKB and testing on SensOrPass), and (iii) transfer learning where models pretrained on UKB are fine-tuned on SensOrPass using repeated stratified cross-validation

Analysis	Train and Test on SensOrPass data				Sample type transfer Train on UKB and Test on SensOrPass data								Transfer learning Pretrain UKB & Fine-tune SensOrPass			
					Training: UKB				Training: UKB + Synthetic Cases				Fine-tuned Model			
Model	AUC	F1-Score	Precision	Recall	AUC	F1-Score	Precision	Recall	AUC	F1-Score	Precision	Recall	AUC	F1-Score	Precision	Recall
Logistic Regression - elasticnet	0.86	0.72	1.0	0.57	0.24	0.58	0.4	1.0	0.49	0.58	0.41	1.0	-	-	-	-
Linear Discriminant Analysis (LDA)	0.85	1.0	1.0	1.0	0.22	0.58	0.4	1.0	0.53	0.58	0.41	1.0	-	-	-	-
K-Nearest Neighbors (KNN)	0.87	0.92	1.0	0.86	0.62	0.58	0.4	1.0	0.62	0.58	0.4	1.0	-	-	-	-
Decision Tree Classifier (CART)	0.79	0.86	0.78	1.0	0.55	0.58	0.4	1.0	0.48	0.56	0.4	1.0	-	-	-	-
Gaussian Naive Bayes (NB)	0.84	0.86	0.86	0.86	0.4	0.58	0.41	1.0	0.5	0.0	0.0	0.0	-	-	-	-
Support Vector Machine (SVM)	0.92	0.86	0.86	0.86	0.43	0.58	0.4	1.0	0.58	0.54	0.51	0.59	-	-	-	-
XGBoost	0.88	0.92	1.0	0.86	0.66	0.61	0.47	0.89	0.69	0.63	0.54	0.75	-	-	-	-
CNN-Raw	-	-	-	-	0.77	0.66	0.71	0.62	-	-	-	-	0.56 ± 0.12	0.40 ± 0.16	0.62 ± 0.19	0.42 ± 0.20
**CNN-Synth**	-	-	-	-	-	-	-	-	**0.88**	**0.79**	**0.85**	**0.75**	0.75 ± 0.11	0.58 ± 0.10	0.87 ± 0.06	0.49 ± 0.10

Values in bold indicate the performance of the best-performing model (CNN-Synth).

To further investigate the clinical relevance of these correct predictions, we examined the cancer stage distribution of the 52 true-positive cases in CNN-Synth and 43 true positives in CNN-Raw. As shown in Fig. 2c, both CNN-Raw and CNN-Synth were able to detect cancers across a range of stages (I–IV), including early-stage disease. At all stages, CNN-Synth demonstrated greater sensitivity, and misclassification rates were highest for both models at Stage III.

Fine-tuning of the pretrained CNN models on the SensOrPass cohort was performed using repeated stratified cross-validation (5 folds repeated 3 times). As shown in Table 2, the CNN-Synth model achieved the best performance in the sample type transfer setting, outperforming all other approaches. When fine-tuning was applied, CNN-Synth maintained a higher AUC compared with CNN-Raw.

### Comparison to non-neural-network cancer classifiers

We compared the performance of our CNN models with a selection of non-neural-network models (see Methods Section “Non-neural-network protein-based cancer classifiers”) according to two different scenarios (Fig. 1). In the sample type transfer scenario (training in UKB and testing in SensOrPass - left panel shown in Fig. 1), the CNN models consistently outperformed these non-neural-network models (Fig. 2a and Table 2). After the CNN models with AUCs of 0.77 and 0.88, XGBoost has the next best performance at AUC = 0.66, followed by KNN at AUC = 0.62 and CART at AUC = 0.55.

To ensure a fairer comparison, we additionally trained the non-neural network models on the combined UKB and synthetic cases, and the results are presented in Table 2. Incorporating synthetic data led to moderate improvements across these models, with XGBoost achieving an AUC of 0.69, LDA 0.55, and SVM 0.58. However, CNN-Synth continued to outperform all non-neural network models, underscoring the advantage of the CNN architecture in leveraging synthetic data for improved generalization across domains.

In the training and testing scenario carried out from scratch (i.e., entirely within the SensOrPass dataset under cross-validation, right panel of Fig. 1), performances were generally much higher overall (Supplementary Fig. 2a–g). In fact, average AUCs were as high as 0.92, slightly higher than CNN-Synth in the sample type transfer scenario. However, these performances should be interpreted with caution due to the high variance in performances across the 10 cross-validation folds (Supplementary Fig. 2h). The high variance suggests over-fitting due to small sample size. A direct comparison to a CNN model in this scenario was not made because the dataset was too small to train a CNN.

### Model interpretation using Shapley values

**Fig. 3:**
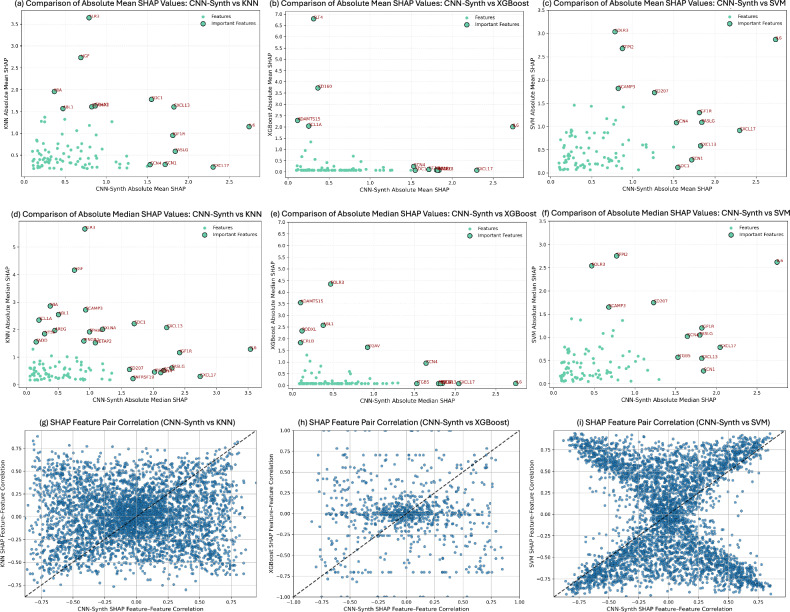
SHAP analysis for best performing models from the sample type transfer approach. **a**–**c** Feature importances (mean absolute Shapley values) for CNN-Synth versus other non-neural-network models. **d**, **e** Feature importances (median absolute Shapley values) for CNN-Synth versus other non-neural-network models. **g**–**i** Pairwise feature Shapley value correlations of CNN-Synth versus those of KNN (*r* = 0.013), XGBoost (*r* = −0.173) and SVM (*r* = −0.024). Lower correlation values indicate differences in feature attribution patterns across models, suggesting varying degrees of interdependence in learned protein representations. SHAP SHapley Additive exPlanations, KNN k-nearest neighbors, XGBoost eXtreme gradient boosting, SVM support vector machines.

The mean Shapley values in SensOrPass highlight the relative importance of salivary proteomic biomarkers for HNC detection within each trained model. We can therefore compare models by comparing their Shapley values for each protein. Figure 3a–i illustrates comparisons between CNN-Synth and best performing non-neural-network models, XGBoost, KNN and SVM. For the CNN-Synth, proteins such as IL6, CXCL17, CXCL13, IGF1R, and FASLG were identified as the top five contributors, consistent with their established roles in cancer biology. Although there is some agreement with other models, correlations of Shapley values between CNN-Synth and the other models are low (Pearson’s *R* < −0.02). To assess robustness, we quantified the correlation between SHAP values for CNN-Raw (AUC = 0.75) and CNN-Synth (AUC = 0.88). Spearman and Pearson correlations were *ρ* = 0.49 (*p* < 0.001) and *r* = 0.25 (*p* = 0.015), respectively, with top-10 and top-20 feature overlaps of 30% and 40%. While CNN-Raw identified a partly different set of important proteins, key features such as CXCL13 and CEACAM1 were consistently highlighted across both models. These results indicate that synthetic augmentation enhanced model performance while preserving core predictive biological signals, demonstrating stable underlying patterns despite differences in training data.

Pair-wise correlations of Shapley values for pairs of proteins within a given model indicate the extent to which the model captures interactions between the proteins. Figure 3g–i compares the pair-wise correlations for CNN-Synth to the best-performing non-neural-network models. Overall, the interactions captured by CNN-Synth differ markedly from those captured by XGBoost and KNN. According to the Wilcoxon rank-sum test, CNN-Raw exhibits an extremely tight distribution centered near 1.0, indicating highly homogeneous feature rankings that suggest overfitting to specific training set patterns (Supplementary Fig. 3). In contrast, CNN-Synth shows a broader distribution (median ≈ 0.25) spanning correlations below 0.4, reflecting greater diversity in learned protein interactions while maintaining meaningful biological signal. CNN-Synth exhibits significantly stronger correlations between protein features than XGBoost (*p* = 1.75 × 10^−34^) and KNN (*p* = 2.33 × 10^−74^), while no significant difference was observed compared to SVM and CNN-Raw (*p* = 1.0). Although KNN shows a more similar range of correlations, its values remain closer to zero than those of CNN-Synth.

Functional enrichment of the top 20 SHAP-ranked proteins in CNN-Synth revealed significant over-representation of GO ontology terms and KEGG pathways (Supplementary Fig. 4a–c). Enriched terms and pathways were consistent with shared roles in cancer progression, specifically related tumor growth and development. For example, biological processes included morphogenesis, immune cell differentiation, vasculature development. Similarly, enriched molecular functions include extracellular matrix remodeling and intercellular signaling. KEGG pathways included proteoglycans in cancer, PI3K-Akt signaling, and Ras signaling.

## Discussion

In this study, we explore the potential of peripheral protein abundance for cancer detection across tissues. To overcome the challenge of limited sample sizes typical of rare cancer outcomes, we employ deep learning approaches that combine generative models with transfer learning techniques. Specifically, we train deep learning models in a large pan-cancer population with VAE-generated synthetic cancer protein profiles to mitigate class imbalance followed by transferring the resulting model, called CNN-Synth, to a smaller case-control study for performance evaluation. We report evidence that both sample type transfer and synthetic data generation may provide independent improvements in classification performance.

Despite the success of deep learning models at solving a wide variety of general imaging and language processing problems, they have only recently been proposed for early detection of disease using omic-scale data^24–26^. The requirement of large training datasets has previously prevented such applications. However, the recent generation of molecular datasets for large, population-based cohort studies like UKB has made it feasible to investigate their ability to model general molecular patterns that can be transferred to limited target datasets enriched with cases of rare diseases. We provide evidence that, indeed, deep learning with sufficient training data can outperform non-neural-network approaches. In particular, we show that a CNN pan-cancer classifier trained in protein data derived from blood plasma generalizes well to protein data derived from a different cell type, saliva, outperforming non-neural-network models trained either from scratch or within a sample type transfer context. The model’s strong performance on saliva, despite being trained on plasma, perhaps reflects shared systemic protein signatures rather than tissue-specific effects. Multiple methods (e.g., flow cytometry^27,28^ and single-cell RNA-seq^29^) have shown that saliva contains significant proportions of cell types present in blood including erythrocytes, leukocytes, and lymphocytes.

Several studies have explored the potential of UKB proteomic data for early cancer detection. For instance,^30^ used UKB proteomic data to identify biomarkers for colorectal cancer, employing non-neural-network models such as LASSO and XGBoost. While their work demonstrates the potential of proteomics for early detection, it is limited to a single cancer type and does not explore sample type generalizability or deep learning. In contrast, our study presents a proof-of-concept framework combining sample type transfer, synthetic data augmentation, and explainable AI to improve performance and interpretability across tissue types.

Further, by application of generative deep learning models to measurements performed in sufficiently large sample sizes, it is feasible to model underlying distributions and interactions in protein abundance datasets that can be used to generate realistic synthetic protein profiles. Specifically, we train a VAE, a particular class of generative neural net model, on a limited number of protein measurements in a large population cohort and observe that synthetic protein abundance profiles sampled from this model closely matched the marginal distributions of the original training data. Although the VAE accurately reproduced these marginal distributions, it did not fully capture the multivariate dependence structure of the real cohort. This is consistent with the tendency of VAEs to generate samples with reduced variance and weaker correlations, even in moderately sized proteomic datasets^31^. As a result, synthetic samples remain distinguishable from real ones under multivariate classification, despite strong feature-level alignment. Importantly, this limitation does not affect their intended use in our study: augmentation for class-imbalance correction primarily requires realistic marginal representations rather than perfect reconstruction of higher-order relationships. Future work may investigate alternative generative frameworks, such as *β*-VAEs or flow-based models^32,33^, to better capture protein-protein dependency patterns. We confirmed the value of synthetic protein profiles generated by VAE by including them when training CNN-Synth. Their inclusion in training improved classification performance substantially (from AUC = 0.77–0.88 and F1-score = 0.66–0.79).

Our study contributes to the growing body of applied studies^11,20^ that aim to solve the class imbalance problem in omics data using generative deep learning models.^20^ incorporates GANs to simulate and augment synthetic data whereas^11^ uses VAE. VAEs have shown strengths in specific generative tasks that benefit from latent space representations, such as drug design^34^ and dimensionality reduction of high-dimensional omics data^15^. While GANs are more commonly applied in generating realistic medical images^35^ or creating synthetic EHR data for privacy preservation^36^, VAEs excel in applications that require a more structured probabilistic framework for learning underlying data distributions. Like our study, both studies^11,20^ use transfer learning, but ours differs by not requiring any re-training or fine-tuning of pre-trained models in target data. For example,^11^ fine-tunes the pre-trained TCGA model on each cancer subtype.

Approaches based on deep learning have long been criticized for not being interpretable^11,15^. Fortunately, recent methods for understanding complex model performance have improved greatly, including SHAP^23^ and LIME (Local Interpretable Model-agnostic Explanations)^37^. We selected SHAP to investigate our CNN-Synth model because it provides both global and local feature importance using Shapley values as opposed to the local-only by design behavior of LIME. Our SHAP approach prioritizes consistency and accuracy of interpretation over flexibility preferred by LIME.

Shapley values from our CNN-Synth cancer classifier highlighted roles for IL6, CXCL13, and CXCL17 as key biomarkers, adding to existing evidence supporting their potential roles in cancer pathogenesis and their utility in early cancer detection^38–40^. These findings demonstrate the potential of deep learning models for biomarker discovery and personalized cancer diagnostics, even in the context of limited data availability

Comparison of Shapley values between CNN-Synth and non-neural network models highlighted few similarities in the contribution of individual proteins to predictions. Further comparison of correlations between Shapley values for pairs of proteins aimed to illustrate the contributions of protein interactions to model predictions. Not surprisingly, we observe that a linear model like SVM utilizes little or no interaction information for prediction. XGBoost, by contrast, captures limited interaction information, reflecting its tree-based architecture that models weak non-linearities. However, the overall correlation strengths remain significantly lower than those of CNN-Synth (*p* = 1.75 × 10^−34^, Wilcoxon rank-sum test). Among the non-neural network models, KNN showed the most overlapping pattern of SHAP correlations with CNN-Synth, consistent with its flexible, instance-based learning mechanism. Nonetheless, the distributions remained statistically distinct (*p* = 2.33 × 10^−74^), reflecting systematic differences in how the two models represent protein interactions. In contrast, the linear SVM model showed near-zero pairwise correlations for most proteins, resulting in a non-significant test (*p* = 1.0) due to its inability to capture interaction effects. When we directly examined CNN-Raw results in the (Supplementary Fig. 3), it was evident that CNN-Synth consistently assigned strong, coordinated attributions across interacting protein pairs, producing dense clusters of high correlation that were largely absent in the other models. CNN-Raw correlations appear excessively strong and less distributed, suggesting potential overfitting to the training data compared to the more balanced attributions of CNN-Synth. Together, these results suggest that CNN-Synth captures more interdependent and non-linear feature attributions, better reflecting complex biological dependencies between proteins. These findings suggest that the CNN captures more complex interactions, and this improved representation of underlying interactions may be driving its improved classification performance.

We have shown that the most prominent proteins in CNN-Synth, as identified by Shapley analysis, are known to play key roles in biological processes, molecular functions, and pathways that have been implicated in cancer progression, particularly tumor growth and development^41^. Although CNN-Synth was not trained and has not been evaluated in tumor tissue directly, it would not be surprising if consistent signals appear in peripheral tissues like blood and saliva given their direct contact with HNC tumors, either as a passive effect of proximity or a systemic response. It remains for future studies to investigate the exact nature of these relationships in further detail.

Our study has several limitations. Although our CNN performs well in data derived from saliva (SensOrPass) after having been trained on data derived from blood (UKB), we suspect that performance would have been better if training and testing tissues had been the same. Our study nonetheless highlights the important and perhaps surprising overlaps in biomarkers between tissue types that were uncovered only by a deep learning model rather than simpler, more traditional machine learning models. Performance also likely suffered due to low numbers of HNC (14% of cancer cases) available in UKB for training. We therefore chose to train the model to differentiate between cases from any cancer and controls. In spite of this limitation, our study highlights important and surprising similarities in biomarkers that appear to be shared between many cancers. However, broad conclusions about similarities between cancers are limited by the fact that model testing was restricted to a single, small head and neck cancer (HNC) dataset. Although the SensOrPass cohort is relatively small and ethnically homogeneous, the use of an independent dataset for testing minimizes cohort-specific bias and supports the robustness of the model. However, since both the training and testing cohorts include only White British participants and no additional replication datasets are available, the generalizability of our models to other ethnicities or genetic ancestries remains uncertain. Future validation using larger, ethnically diverse, and multi-cancer datasets will be essential to confirm the generalizability of the proposed approach across populations and tissue types. We acknowledge that CNN-Synth is unlikely to be fully optimized. For example, we did not explore whether similar performance could have been achieved with a simpler neural net modeling strategy (e.g., multi-layer perceptron) or by using ensembling to balance the higher but more variable accuracy of non-neural-network models with the more stable performance of CNN. We hope that our study will motivate future studies to explore use of this approach for pan-cancer early detection. Our study investigated only the measurement of a modest number of proteins (92). Model performances may have been improved if measurements of other proteins had been available, as well as other molecular measurements such as gene expression, DNA methylation, and/or metabolite levels. It is important to note that the 92 proteins measured in SensOrPass were drawn from a pre-selected panel of proteins known to be relevant to cancer diagnosis. Although this small selection may have omitted predictive proteins, it may also have ensured specificity in the CNN architecture and improved the interpretability of the resulting CNN model.

In conclusion, our results provide a proof-of-concept that combining deep learning, sample type transfer from large-scale datasets, and synthetic sample generation can partially overcome challenges in disease detection associated with limited sample sizes, tissue differences, and class imbalance. This approach lays the groundwork for future studies aimed at improving early-stage cancer classification and expanding clinical applicability.

## Methods

### Study populations: UK Biobank (UKB)

UKB is a population-based cohort with data collected from over 500,000 adult participants aged between 40 and 69 years that were recruited between 2006 and 2010 in the UK. It includes extensive phenotypic, clinical, and multi-omic measurements. Our study used data from a subset of *N* = 54,219 UKB participants selected as part of the UKB Pharma Proteomics Project^8^, hereafter referred to as UKB. These participants had 2941 plasma protein analytes representing 2923 unique proteins measured by the Olink Explore 3072 Protein Extension Assay (Uppsala, Sweden). Comprehensive details on proteomic data generation, along with normalization and quality control procedures, have been published^8,42^. Protein levels were reported and analyzed as normalized protein expression (NPX) on the log base 2 scale. For compatibility with the measures available in our test set, we restricted all analyses to the 92 proteins that were also available on the Olink Oncology II panel used in the SensOrPass cohort, which was designed to measure proteins previously identified as associated with cancer.

Information on cancer incidence for UKB was obtained via the cancer register. We identified *N* = 13,208 cancer cases as those who had “reported occurrences of cancer” and “date of cancer diagnosis” for all instances in the cancer registry (Table 1 and Supplementary Table 1). The cases included a wide range of malignancies, including 14% with malignancies of the lip, oral cavity, pharynx, or oropharynx (Supplementary Fig. 7). All other remaining participants were included as controls. Of these 13, 208 cases, *n* = 8262 where diagnosed after sample collection and *n* = 4945 where diagnosed before sample collection, the time-to-diagnosis distributions are shown in the Supplementary Fig. 5. These counts are computed using the variable “Date of cancer diagnosis” and “Sample collection sign-off timestamp”.

UK Biobank has Research Tissue Bank approval from the North West Multi-centre Research Ethics Committee (Reference:16/NW/0274), allowing researchers with valid access to perform research without need for further ethical approval. All participants gave consent for their de-identified data to be used for health-related research that is in the public interest.

### Study populations: SensOrPass head and neck cancer case-control study

SensOrPass is a *N* = 156 participant head and neck cancer (HNC) case-control study recruited from the south west of England with the aim of identifying saliva protein biomarkers of HNC cancer that would be suitable for passive monitoring with electrochemical sensors. *N* = 14 treatment-naive HNC cases were recruited at the Royal Devon University NHS Foundation Trust and Hampshire Hospitals NHS Foundation Trust. An additional *N* = 50 cases were selected from among the participants in the Head *&* Neck 5000 cohort with cancer (stages I–IV) at diagnosis with available saliva samples (Fig. 2c)^43,44^. A *N* = 92 control group was recruited from healthy volunteers of the Exeter 10,000 cohort^45^. The abundances of 92 proteins were measured in saliva samples using the Olink Oncology II panel (Table 1 and Supplementary Table 2).

The SensOrPass study was approved by the NHS North West - Haydock Research Ethics Committee (Ref: 22/NW/0351; approval date: 31 January 2023). Informed consent was obtained from all participants prior to participation, including consent for the collection and analysis of saliva samples and associated data for research purposes.

### Variational autoencoder (VAE) of cancer-specific protein abundance

**Fig. 4:**
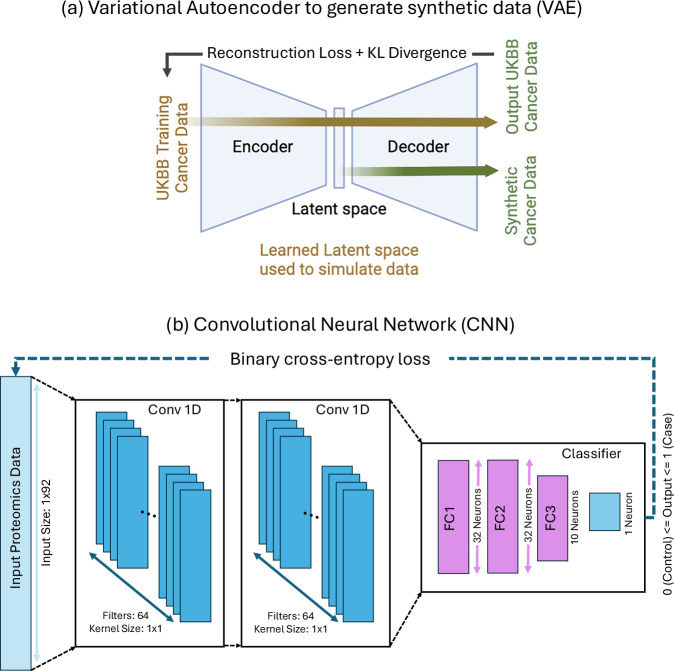
Network architectures of VAE and CNN models. **a** Encoder consists of four fully connected layers of size 92, 50, 25, and 10. Decoder consists of a symmetric set of four fully connected layers of size 10, 25, 50, and 92. **b** Both CNN-Synth and CNN-Raw include 2 convolutional layers with 64 filters each following a classifier consisting of 3 fully connected layers of sizes 32, 32, and 10. Output consists of a single neuron with a sigmoid activation function. The model is trained using the binary cross-entropy loss function.

Towards the aim of simulating synthetic cancer-specific protein profiles, we trained a VAE from UKB cancer cases using the 92 proteins from Olink Explore 3072 that were also available on the Oncology II platform in the SensOrPass validation data. The VAE architecture consisted of a protein input layer that is passed to an encoder with three fully connected layers of decreasing size–50, 25, and 10 neurons, respectively–to encode the input data into a lower-dimensional latent space (Fig. 4a). Synthetic observations can be sampled from the encoder-generated latent space via a decoder of roughly symmetrical architecture, starting with a fully connected layer of size 10, followed by layers of 25 and 50 neurons and final output layer corresponding to the 92 reconstructed proteins. A Tanh activation function was used for every layer except the output layer. The model was optimized using the Adam optimizer and trained with a learning rate of 10^−6^.

To train the VAE, we defined a total VAE loss function to balance reconstruction accuracy with regularization of the latent space, allowing the model to learn meaningful representations of proteomic data while preventing overfitting. The total VAE loss is made up of two components: (1) the reconstruction loss and (2) the KL divergence loss.

For reconstruction loss, which quantifies the difference between the original input and its reconstruction by the decoder (Eq. (1)), we specified a mean squared error (MSE) loss function:

1 $${{\mathcal{L}}}_{\mathrm{reconstruction}}=\parallel \,x-\widehat{\,x}{\parallel }^{2}$$

For KL divergence (Eq.: (2)), we specified how much the learned latent distribution *q*(*z*∣*x*) diverges from a standard normal prior distribution ${\mathcal{N}}(0,I)$:

2 $${{\mathcal{L}}}_{\mathrm{KL}}={D}_{\mathrm{KL}}(q(z| x)\parallel p(z))=\frac{1}{2}\mathop{\sum }\limits_{j=1}^{J}(1+\log ({\sigma }_{j}^{2})-{\mu }_{j}^{2}-{\sigma }_{j}^{2})$$

where *μ* and *σ* are the mean and standard deviation vectors from the encoder’s output, and *J* is the dimensionality of the latent space.

Total VAE loss combined both (Eqs. (1) and (2)) as shown below:

3 $${{\mathcal{L}}}_{\mathrm{VAE}}=\parallel x-\widehat{x}{\parallel }^{2}+\frac{1}{2}\mathop{\sum }\limits_{j=1}^{J}(1+\log ({\sigma }_{j}^{2})-{\mu }_{j}^{2}-{\sigma }_{j}^{2})$$

VAE training aimed to minimize (Eq. (3)) using all available UKB cancer cases (*N* = 13,208). Training progress was assessed by closely monitoring the training loss (Supplementary Fig. 6a) and was halted when the reconstruction loss plateaued at 50 epochs.

### Protein-based CNN cancer classifiers (CNN-Raw and CNN-Synth)

In UKB data, we trained two protein-based CNN cancer classification models^30,46,47^, CNN-Raw and CNN-Synth. Both models were trained using UKB plasma protein abundance data to distinguish between pan-cancer cases and controls. Training was identical for both models, except that the CNN-Synth training dataset included 10,000 additional cancer-specific protein profiles synthesized by the VAE described above; these synthetic profiles were generated solely from UKB training cases and were not included in the validation or testing datasets, ensuring full independence and preventing data leakage. These model inputs were presented as a 1 × 92 input vector of features, corresponding to those available in both UKB and SensOrPass, and the model output represented estimated probability of cancer.

We specified a network architecture consisting of two 1D convolutional layers, each using 64 filters with a kernel size of 1 × 1 (Fig. 4b). The input proteomic profile, consisting of 92 protein abundance measurements, is represented as a tensor of shape (1, 92), where each protein corresponds to a feature channel. In this representation, the 1 × 1 convolution operates across channels and performs a learnable linear projection that mixes information across protein features. Although the 1 × 1 convolutions resemble operations in a multi-layer perceptron (MLP), they provide a structured mechanism for channel-wise feature weighting within a convolutional framework. Stacking multiple pointwise convolutional layers with nonlinear activation functions enables the model to capture nonlinear interactions among the 92 protein features while maintaining a relatively parameter-efficient architecture compared with conventional fully connected layers. Each convolutional layer *l* applied filters to the input through the following operation:

4 $${{\bf{z}}}_{j}^{(l)}=\mathop{\sum }\limits_{i=1}^{92}{{\bf{x}}}_{i}* {{\bf{w}}}_{i,j}^{(l)}+{b}_{j}^{(l)}$$

where *j* is the number of filters, **x**_**i**_ is the input of each layer, ${{\bf{w}}}_{i,j}^{(l)}$ are the weights of the filter *j* and the input *i*, ${{\bf{b}}}_{j}^{(l)}$ is the bias for each filter, and * denotes the 1D convolution operation. The length of the output feature map for each filter is computed as:

5 $${L}_{\mathrm{out}}=\left\lfloor \frac{{L}_{\mathrm{in}}+2P-K}{S}\right\rfloor +1$$

where *L*_in_ is the length of the input vector, *K* is the kernel size (1 in our case), *P* is the zero-padding size (typically 0 unless otherwise specified), and *S* is the stride with which the kernel moves across the input vector. These convolutional layers are followed by a ReLU activation function before feeding into three fully connected layers intended to integrate and refine the extracted features for binary classification. The first and second fully connected layers contain 32 and 10 neurons, respectively. The final output is specified by a sigmoid function with output values between 0 (control) and 1 (case), which are interpreted as probabilities.

Training aimed to minimize the binary cross-entropy loss function (Eq. (6)), which measures how well the model’s predicted probability *p* aligns with the true label *y*^48^:

6 $$L=-(y\log (p)+(1-y)\log (1-p)).$$

All models were trained using early stopping, where training was halted if the validation loss did not improve for a predefined number of consecutive epochs (i.e., 15), in order to prevent overfitting and ensure generalization. For the CNN-Synth model, early stopping occurred at epoch 50 (see Supplementary Fig. 6b).

To assess whether adapting this pretrained model to the target tissue (saliva) improves performance, we performed a transfer-learning experiment using the SensOrPass. The pretrained CNN on the UKB dataset and the resulting weights were used to initialize the model. We then evaluated target-domain adaptation using repeated stratified cross-validation on the SensOrPass dataset (5 folds, repeated 3 times) to ensure robust performance estimates given the limited sample size. In each fold, 80% of the SensOrPass samples were used for training and 20% were held out for testing. Within the training portion, a stratified subset (15%) was further reserved for validation and early stopping. During fine-tuning, the convolutional layers and early fully connected layers (FC1, FC2, and FC3 as shown in Fig. 4b) were initially frozen to preserve the pretrained feature representations, and only the classification head was trained on the HNC data. Subsequently, the upper dense layers were unfrozen and the model was fine-tuned with a lower learning rate to allow limited adaptation to the target domain while reducing the risk of overfitting.

### Non-neural-network protein-based cancer classifiers

To compare the performance of our protein-based generalized CNN cancer discrimination model against a wide range of alternative approaches, we trained a diverse library of non-neural-network methods for comparison to the CNN models described above (Section “Protein-based CNN cancer classifiers (CNN-Raw and CNN-Synth”)). These span various algorithmic families with differing assumptions and inductive biases. Specifically, we evaluated logistic regression with L1/L2 regularization to mitigate overfitting and enhance generalization (LR-elasticnet), linear discriminant analysis (LDA), k-nearest neighbors (KNN), decision trees (CART), Gaussian naïve Bayes (NB), support vector machines (SVMs), and eXtreme Gradient Boosting (XGBoost), which are well-suited for handling high-dimensional and potentially sparse feature spaces. To ensure consistency and comparability, we tuned the hyperparameters of each model using grid search with 10-fold cross-validation, selecting the best parameters based on accuracy. For LR-ElasticNet, we set *C* = 0.01 and l1 ratio = 0.5; for LDA, the solver was set to “lsqr“ with shrinkage = “auto”; for KNN, we used 7 neighbors with distance-based weighting; for CART, the maximum depth was unrestricted and the minimum samples per split was 2; for SVM, a linear kernel was used with gamma = “scale”, probability = True, *C* = 1, and a maximum of 5000 iterations; finally, for XGBoost, the model was fit with a maximum tree depth of 6, learning rate of 0.1, subsample of 0.7, column sampling by tree of 0.6, scale pos weight of 0.7, and regularization parameters *λ* = 1 and *α* = 0.9.

Non-neural-network model performances were evaluated in two different training-testing scenarios. In the first scenario, models were trained in UKB as well as on a combined dataset of UKB and synthetic cases, and subsequently tested in SensOrPass, similarly to the CNN models described above (sample type transfer approach - left panel shown in Fig. 1). Here, sample type transfer refers to the direct application of models trained on a source dataset (UKB) to a target dataset (SensOrPass) without any fine-tuning or retraining (following the general principle of zero-shot learning^49^, allowing assessment of how well patterns learned from the source domain generalize to unseen target data. In the second scenario, models were trained from scratch on SensOrPass data and performances evaluated within the context of 10-fold cross-validation using only SensOrPass data (right panel shown in Fig. 1).

We employed the precision-recall curve to determine optimal classification thresholds. For each model, we selected the threshold that maximizes the F1-score, which represents the harmonic mean of precision and recall. This approach ensures balanced performance between false positives and false negatives.

### Classification performance

We evaluated classification performance by confusion matrices, precision (how many predicted positives are actually cancer cases), recall (how many actual cancer cases were correctly identified), and F1-score (the harmonic mean of precision and recall). We used Area Under the Receiver Operating Characteristic (ROC) curve (AUC) to summarize the classification performance for each model across the range of threshold values.

### Model Interpretation Using Shapley values

To interpret the role of individual proteins in cancer classification models, we used SHapley Additive exPlanations (SHAP)^23^ within the SensOrPass dataset to estimate feature importance values. This was applied to both deep learning (CNN-Synth and CNN-Raw) and non-neural-network models (KNN, XGBoost, and SVM) to enable a consistent comparison across these models.

For each model, the corresponding SHAP explainer was selected according to its architecture: KernelExplainer for the SVM and KNN, TreeExplainer for the XGBoost model, and DeepExplainer for the CNN-Synth. The explainers compute per-sample SHAP values that quantify the marginal contribution of each protein to the model’s prediction relative to an expected baseline output. For the CNN-Synth model, the explainer was initialized with the trained classifier (SVM, XGBoost, and CNN-Synth) and the background dataset (SensOrPass dataset) to estimate expected values. SHAP values were then derived for all correctly classified samples to ensure that the feature importance interpretation reflects reliable model behavior.

For each sample, SHAP generates contribution scores across all possible output classes (control and case). To obtain interpretable global feature rankings, we considered the SHAP values corresponding to the predicted class only and summarized them by computing the mean and median absolute SHAP value of each protein across all samples (Fig. 3a–e). Both methods produced nearly identical results, indicating that the feature importance rankings are robust to outliers. These aggregated scores were visualized, highlighting the most influential proteins driving the model’s cancer detection performance. To assess the biological relevance of the identified markers, we performed functional enrichment analysis using Enrichr on the top 20 SHAP-ranked proteins, with the UKB plasma proteome used as background. The Enrichr analysis of the top SHAP-ranked proteins showed significant enrichment across GO Biological Process, GO Molecular Function, and KEGG pathways (Supplementary Fig. 7). This approach allows for a transparent understanding of model decisions and provides biologically interpretable insights into the relative contribution of protein markers in distinguishing between cancer and control samples.

### Implementation resources

All analyses were performed in Python versions 3.9. 18. Our deep learning models, VAE (Section “Variational autoencoder (VAE) of cancer-specific protein abundance”) and CNN (Section “Protein-based CNN cancer classifiers (CNN-Raw and CNN-Synth)”), were trained and evaluated using Keras with a TensorFlow backend (version: 2.18.0)^50^. All other machine learning models (Section “Non-neural-network protein-based cancer classifiers”) were implemented and benchmarked for performance in scikit-learn^51^. Finally, feature importance and model explanability was performed using the SHAP Python package^23^.

## Supplementary information

Supplementary Information

## Data Availability

UKB data are available to access by application procedure detailed here: http://www.ukbiobank.ac.uk/using-the-resource/. SensOrPass data, study protocol, and data dictionary are available from the Head and Neck 5000 and Exeter 10,000 research resources. Full application details are available here: https://headandneck5000.org.uk/information-for-researchers/ and https://exetercrfnihr.org/about/exeter-10000/. The code for this study is publicly available on GitHub: https://github.com/MRCIEU/CNN-Synth-cancer-detection-using-deep-transfer-learning-and-data-synthesis. The repository contains the deep learning model for cancer classification using protein biomarkers, trained on UKB datasets. It includes scripts for model training, evaluation, and explainability, as well as the necessary dependencies specified in the requirements.txt file.
