# Supplementary material for: Leveraging population-scale proteomic data with deep learning for head and neck cancer detection in saliva
Source: NPJ Digit Med. 2026 May 2;9:523. doi: 10.1038/s41746-026-02658-7 (PMC13342633; doi:10.1038/s41746-026-02658-7)
Supplement: Supplementary file 1 — Supplementary Information [file 41746_2026_2658_MOESM1_ESM.pdf]

Leveraging a population-scale proteomic dataset with deep learning for head and neck cancer detection in saliva

Supplemental Material

Supplementary Tables and Figures

Table Supplementary Table 1 UKB cohort details

		Missing	Overall	Case	Control
n			53,014	13,208	39,806
Age, mean (SD)		0	56.8 (8.2)	59.9 (7.2)	55.8 (8.3)
Gender, n (%)	Female		28,580 (53.9)	7,050 (53.4)	21,530 (54.1)
	Male		24,434 (46.1)	6,158 (46.6)	18,276 (45.9)
Smoking status, n (%)	Current		5600 (10.6)	1,475 (11.2)	4,125 (10.4)
	Never		28,670 (54.1)	6,430 (48.7)	22,240 (55.9)
	None		61 (0.1)	10 (0.1)	51 (0.1)
	Prefer not to answer		194 (0.4)	51 (0.4)	143 (0.4)
	Previous		18,489 (34.9)	5,242 (39.7)	13,247 (33.3)
Alcohol status, n (%)	Current		48,306 (91.1)	12,132 (91.9)	36,174 (90.9)
	Never		2506 (4.7)	512 (3.9)	1,994 (5.0)
	None		61 (0.1)	10 (0.1)	51 (0.1)
	Prefer not to answer		80 (0.2)	18 (0.1)	62 (0.2)
	Previous		2,061 (3.9)	536 (4.1)	1,525 (3.8)

Table Supplementary Table 2 SensOrPass cohort details

		Missing	Overall	HNC Case	Control
n			156	64	92
Age, mean (SD)		0	63.6 (9.7)	61.0 (8.5)	65.3 (10.2)
Family history of cancer, n (%)	No		22 (14.1)	5 (7.8)	17 (18.5)
	Yes		79 (50.6)	7 (10.9)	72 (78.3)
	Don't know		5 (3.2)	2 (3.1)	3 (3.3)
	None		50 (32.1)	50 (78.1)	
Gender, n (%)	Male		89 (57.1)	54 (84.4)	35 (38.0)
	Female		67 (42.9)	10 (15.6)	57 (62.0)
Smoking status, n (%)	Yes-cigarettes		8 (5.1)	7 (10.9)	1 (1.1)
	Ex-smoker		70 (44.9)	39 (60.9)	31 (33.7)
	Never smoked		78 (50.0)	18 (28.1)	60 (65.2)
Alcohol status, n (%)	No		23 (14.7)	7 (10.9)	16 (17.4)
	Yes		133 (85.3)	57 (89.1)	76 (82.6)
BMI, mean (SD)		0	27.0 (4.8)	27.1 (4.6)	26.8 (4.8)

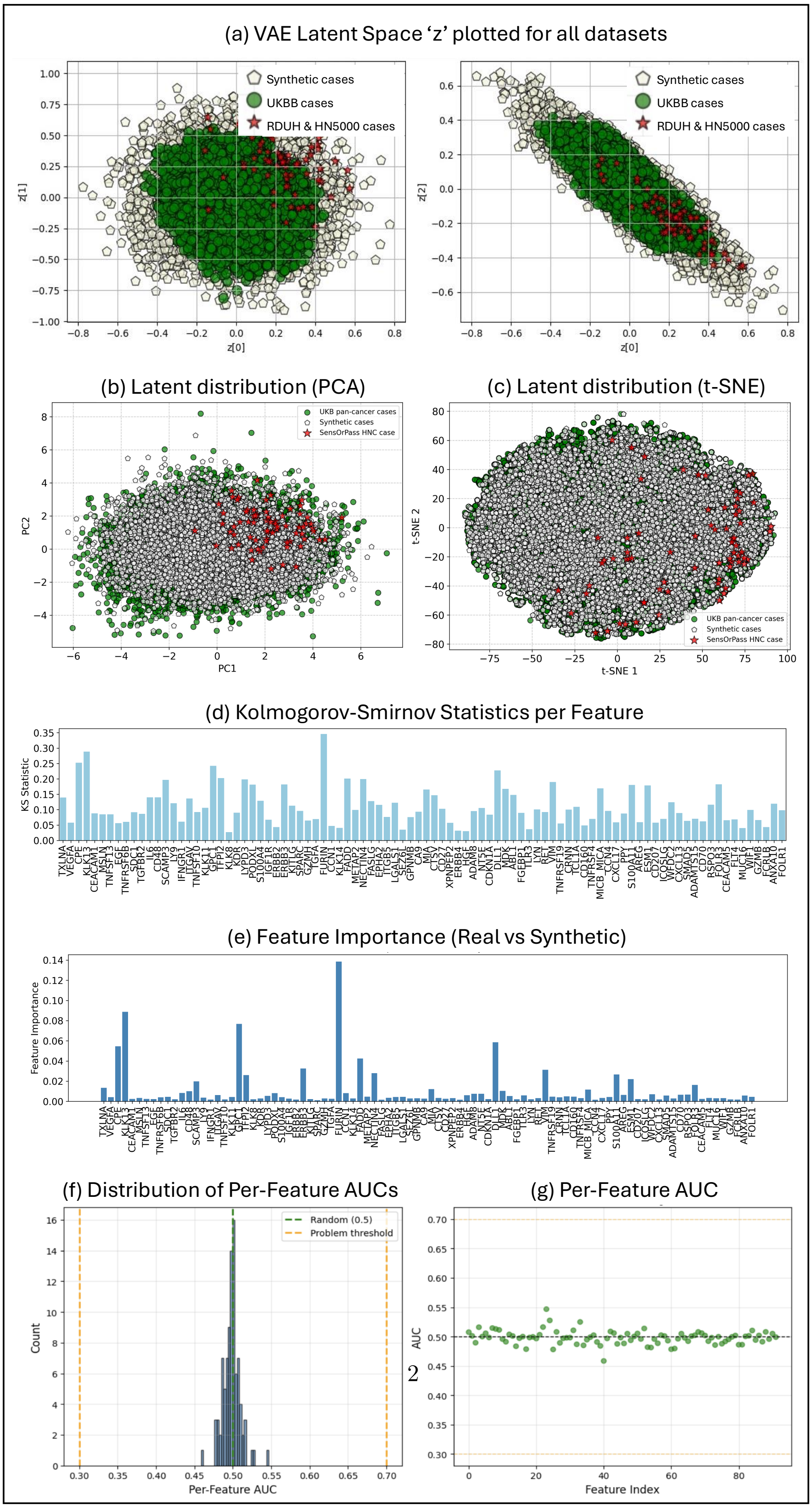

Fig. Supplementary Figure 1 VAE results and comparisons between distributions of real and synthetic cancer cases. **(a)** Visualization of the VAE latent space for UKB, SensOrPass, and synthetic samples. Two pairwise projections of the latent variables are shown: Z_0 vs Z_1 and Z_0 vs Z_2. These projections illustrate the distribution of samples in the learned latent representation.**(b)** PCA of latent distribution. **(c)** t-SNE of latent distribution. **(d)** KS statistics per feature (real vs synthetic). **(e)** Random Forest Feature Importance distinguishing between real and synthetic data. **(f)** Distribution of AUC values obtained from 92 univariate logistic regression models trained to distinguish real from synthetic samples using each protein feature independently. **(g)** Per-feature AUCs plotted by feature index.

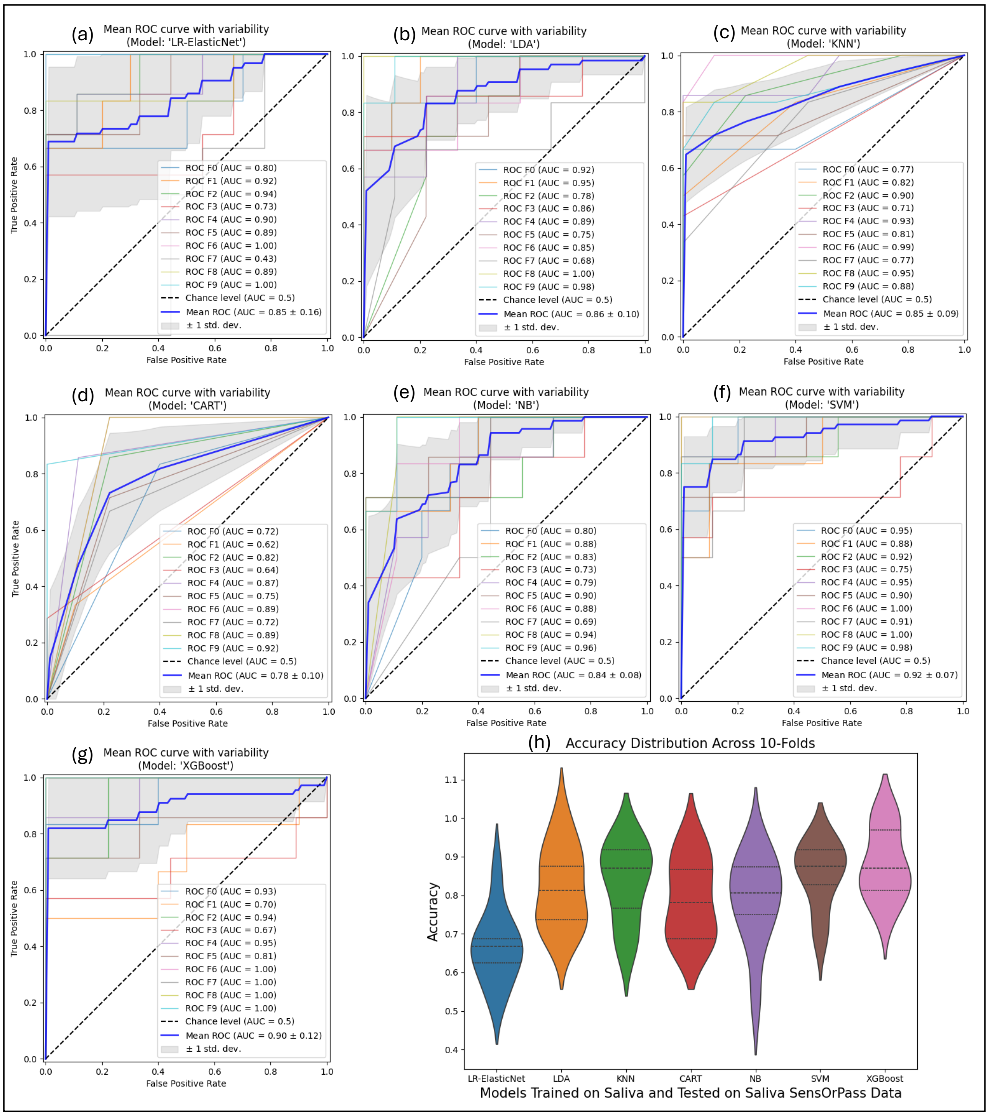

Fig. Supplementary Figure 2 Model Performance When Trained from Scratch. **(a-g)** Shows the ROC plot of non-neural-network models that are trained and tested on saliva proteome data from SensOrPass study. **(h)** Shows the variance of accuracy across the 10 folds of cross-validation for each of these machine learning models.

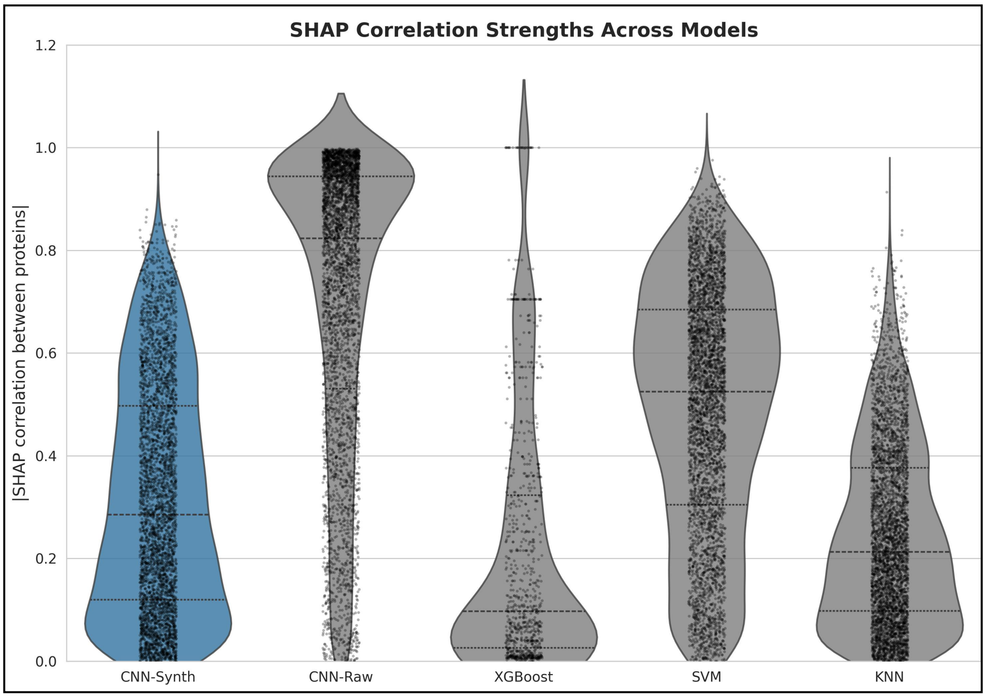

Fig. Supplementary Figure 3 Pairwise statistical comparison of CNN-Synth against CNN-Raw and non-neural-network models (XGBoost, SVM and KNN) trained on UKBB and synthetic data and tested on SensOrPass data. Wilcoxon signed-rank tests show CNN-Synth significantly outperforms KNN ($p = 2.3 \times 10^{-74}$) and XGBoost ($p = 1.7 \times 10^{-34}$), while performing comparably to SVM ($p = 1.0$) and CNN-Raw ($p = 1.0$)

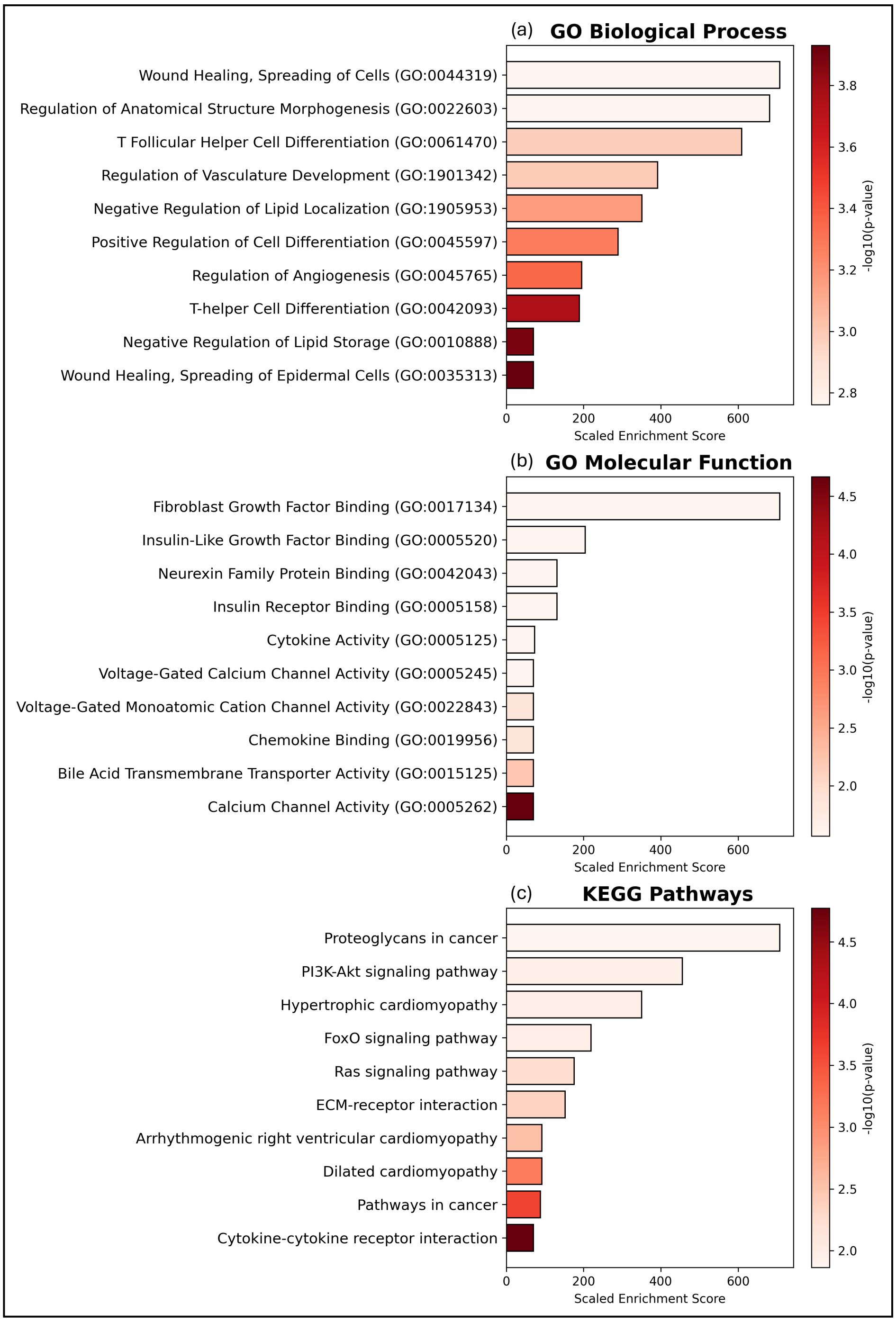

Fig. Supplementary Figure 4 Enrichr analysis of the top SHAP-ranked biomarkers from the SensOrPass saliva model (using the UK Biobank plasma proteome as background). **(a)** GO Biological Processes, **(b)** GO Molecular Functions, **(c)** KEGG pathways. Bars show scaled enrichment scores, with colors reflecting –log10(p-value).

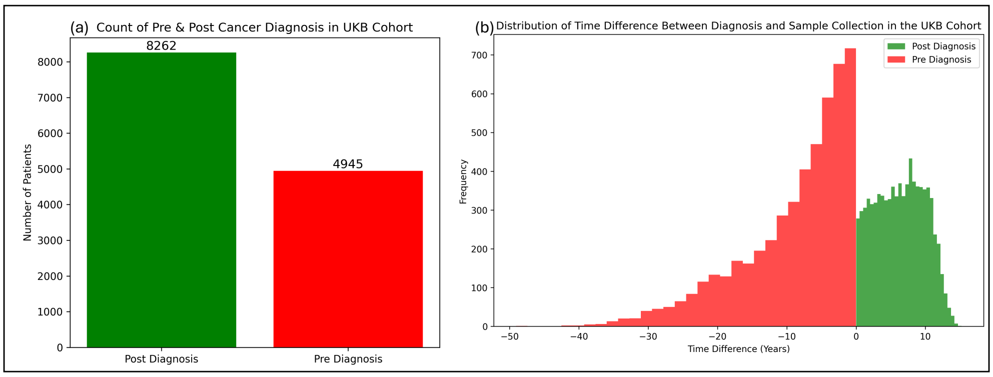

Fig. Supplementary Figure 5 Timing of cancer diagnosis relative to sample collection in UKB. **(a)** Bar plot of cancer diagnosis pre and post sample collection in the UKB data cohort. **(b)** Distribution of change in time when the cancer was first diagnosed and when the samples where drawn for UKB participants. UKB: UK Biobank

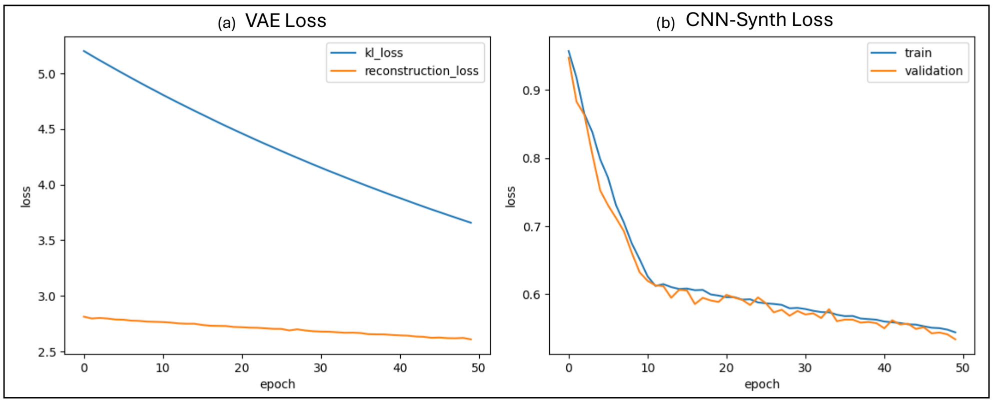

Fig. Supplementary Figure 6 Training loss curves. **(a)** Training loss curves for VAE. **(b)** Training and validation loss curves for CNN-Synth.

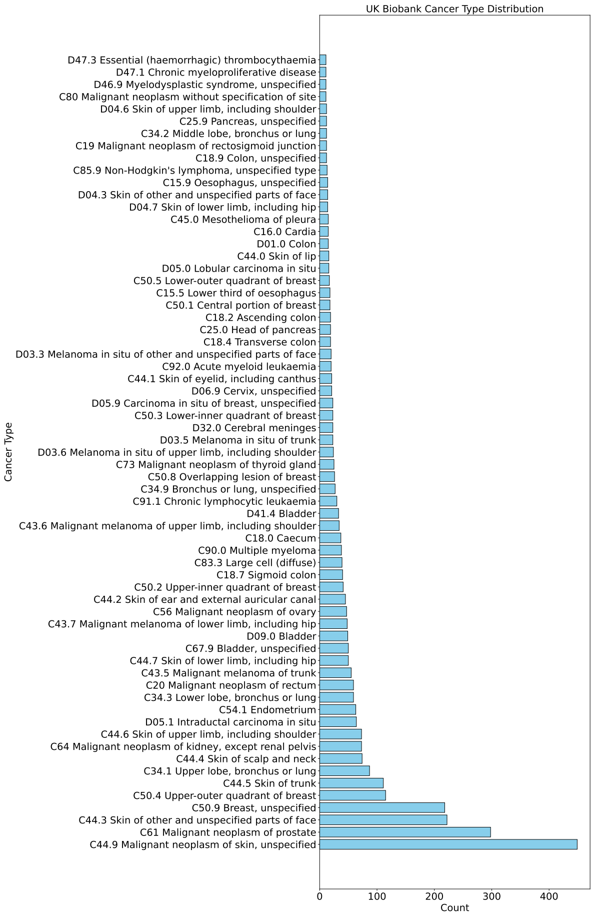

Fig. Supplementary Figure 7 Types of cancer recorded in the UKB dataset.
